# Synovial Parasitosis and Inflammatory Biomarker Profiles in Osteoarthritis: Associations with Host and Therapeutic Factors

**DOI:** 10.1007/s11686-025-01180-2

**Published:** 2025-12-18

**Authors:** Faika Hassanein, Amany I. Shehata, Akram M. Aldawoudy, Inas M. Masoud

**Affiliations:** 1 Faculty of Dentistry, Department of Microbiology & Immunology, Pharos University in Alexandria, Alexandria, Egypt; 2 Department of Tropical Health, High Institute of Public Health, Alexandria University, Alexandria, Egypt; 3 Alex Knee Center, Alexandria, Egypt; 4 Faculty of Pharmacy, Department of Pharmacology and Therapeutics, Pharos University in Alexandria, Alexandria, Egypt

**Keywords:** Synovial, Parasites, TNF, MMP, Osteoarthritic, Corticosteroids, Knee, Osteoarthritis

## Abstract

**Purpose:**

Knee osteoarthritis (OA) is a major cause of chronic disability worldwide, affecting millions and posing a substantial public health burden. It leads to progressive cartilage degeneration, inflammation, and impaired joint function, underscoring the need for early diagnostic biomarkers and insight into contributory factors such as parasitic infections. Recent evidence suggests a connection between knee OA and low-grade intestinal inflammation, as well as alterations in the gut microbiota. We aimed to examine synovial fluid for parasitosis (SP) among osteoarthritic patients (OPs) and to detect associated biomarkers.

**Methods:**

Synovial fluid samples were aspirated and divided into three portions. The first portion was spread to make thin films and stained with various stains. The second portion was centrifuged, and the resulting pellets were inoculated using Jones’ media. The third one was analyzed using the enzyme-linked immunosorbent assay (ELISA) technique to assess Tumor necrosis factor (TNF-α) and matrix metalloproteases-9 (MMP9) biomarkers.

**Results:**

Synovial parasitosis was detected in 57% of OA patients, with Blastocystis sp. being the most prevalent (40%), followed by microsporidia (16%). Corticosteroid-treated (CST) patients had a higher prevalence of SP, while TNF-α and MMP9 levels were elevated in infected compared with noninfected patients. Elevated biomarker levels correlated with infection multiplicity, indicating inflammatory activation.

**Conclusions:**

This study highlights a significant prevalence of SP among OPs (57%), with Blastocystis sp. being the most common parasite. CST-treated patients had a higher SP than those with non-CST. Elevated TNF-α and MMP-9 levels could be biomarkers for identifying patients at risk for disease progression due to SP. Further research should be conducted using molecular techniques, along with studies utilizing antiparasitic treatment among infected OA patients.

**Supplementary Information:**

The online version contains supplementary material available at 10.1007/s11686-025-01180-2.

## Introduction

Knee osteoarthritis (OA) is the most common joint disease worldwide, causing disability in elderly individuals [1]. In 2019, OA affected 32.5 million adults in the U.S. [2], which may increase to 78 million by 2040 [3]. In 2020, its global prevalence was estimated to be 654.1 million individuals aged ≥ 40 years old, with an incidence of 203/10,000 person-years [4]. In Egypt (2019), more than 3 million individuals are affected by OA [5]. It is associated with joint facet cartilage impairment and synovial space inflammation [6]. In chronic stages, joint replacement therapy or other invasive procedures may be needed [7]. It leads to degeneration of all the tissues around the synovial joint, including the ligaments, capsule, metaphyseal bone, and articular cartilage. It also involves chronic, progressive degradation of articular cartilage [8].

Acute OA is a sudden exacerbation of joint pain and inflammation, typically short-lived, lasting from a few days to several weeks, and is often triggered by factors such as joint overuse or minor injuries [9]. In contrast, chronic osteoarthritis is characterized by the progressive and long-term degeneration of joint cartilage, resulting in persistent pain, stiffness, and functional limitations. It develops over many years due to factors like aging, joint wear and tear, or previous joint injuries [10]. OA evolves from asymptomatic early changes to severe degenerative stages characterized by pain, stiffness, and cartilage loss. [11]. The pathogenesis of OA involves three overlapping stages, starting with damage to the extracellular matrix (ECM) of the articular cartilage surface, resulting in cartilage clefts, erosion of the articular surface, fibrillation, subchondral microdamage, and vascular invasion [12, 13]. The degradation of ECM fragments results in the release of proinflammatory and catabolic biochemical markers that produce matrix metalloproteases (MMPs) that subsequently cause degradation of the cartilage extracellular matrix [14]. Bone cysts may appear at the end-stage of OA and are connected to the subchondral surface by a fissure filled with inflammatory fluid, myxoid material, or bone fragments [14, 15].

Physiological changes in synovial fluid (SF) occur in response to trauma, inflammation, and bacterial, fungal, or viral infections. When patients present with acutely painful joints with suspicion of infection, inflammation, or noninflammatory causes of effusion, SF aspiration and analysis are crucial for diagnosing and treatment [16].

Management of knee OA typically begins with conservative interventions and progresses to surgical options [17]. Conservative treatments include physiotherapy, nonsteroidal anti-inflammatory drugs (NSAIDs), and intra-articular injections of hyaluronic acid, corticosteroids, or platelet-rich plasma. B**iomarkers** have been proposed as alternative tools for assessing disease activity [18].

Tumor necrosis factor (TNF) plays a key role in degenerative joint disease [19]. It controls the homeostasis of matrix synthesis and degeneration in articular cartilage with other cytokines. Overproduction of TNF and interleukin-1 (IL-1) leads to matrix degradation, primarily by inducing nitric oxide synthesis and subsequent metalloproteinase production. The pathogenesis of OA is associated with increased TNF production [19, 20]. TNFα-induces MMP-9 secretion in monocytic cells via long-chain acyl-CoA synthetase 1 [21]. Additionally, cytokines, interferon-gamma (IFN-γ), IL-12, and TNF-α can disrupt the intestinal mucosa [22].

The catalytic activity of MMPs results in the degradation of various ECM components. Disrupted MMP activity can cause pathological conditions such as inflammation, arthritis, and cancer [23]. The ECM plays a critical but often overlooked role in infections. Pathogens interact with ECM proteins via various molecules to establish infections, modifying the ECM to evade immune responses (IRs). The ECM undergoes significant changes during infection, influencing immune cell signaling. Immune cells also remodel the ECM by producing various ECM components [24]. Some MMPs are crucial for remodeling the ECM and coordinating the IR during early infection. However, excessive MMP activity during the late stage can lead to tissue damage and potentially death [25]. Unregulated MMPs result in tissue remodeling and fibrosis in chronic pulmonary tuberculosis. Reducing MMP activity has shown benefits in disease models, including *Mycobacterium tuberculosis-*infected rabbits [26] and infections leading to preterm premature rupture of fetal membranes [27]. Dysregulation of MMP activity and expression contributes to the pathogenesis of infectious diseases [28]. Excessive MMP activity induced by infection has been linked to conditions such as sepsis, meningitis, tuberculosis, Lyme disease, and pneumonia [28].

Synovial parasitosis is a rare disease; several intestinal protozoa, including *Entamoeba histolytica*, *Giardia lamblia*, *Toxoplasma gondii*, and *Blastocystis sp.*, are capable of hematogenous or via lymphatic dissemination to extraintestinal sites [29]. Such dissemination may lead to inflammatory or reactive arthritis, as microbial antigens or organisms have been identified in synovial fluid, supporting a potential link between intestinal parasitic infections and joint inflammation in susceptible individuals [30].

Septic arthritis is an infection of the synovium, with the knee being the most commonly affected joint. Recent evidence suggests a connection between OA and low-grade intestinal inflammation, as well as alterations in gut microbiota composition [31]. The gut microbiota—a diverse community of bacteria, fungi, viruses, parasites, and archaea—plays essential roles in metabolism, immunity, and host defense [32–34]. Disruption of the intestinal barrier may allow microbial translocation into the circulation, triggering systemic inflammation and potentially contributing to OA pathogenesis through the so-called “leaky gut” mechanism [35, 36].

To our knowledge, due to the scarcity of studies and the presence of only sporadic reports of parasite dissemination into synovial fluid-such as those by Lee et al., (1990) [37] and Tejera et al., (2012) [38]—and considering that immunosuppressive therapies may contribute to its occurrence, the present study aimed to investigate the possibility of parasitic infection in the synovial fluid of osteoarthritic patients and its correlation with specific biomarker signatures in an epidemiological cross-sectional design.

## Methods

### Study Design and Sample Size

A cross-sectional study was conducted on patients suffering from osteoarthritis attending private osteoarthritic out-clinics at the Center of Orthopedics and Joint Diseases in Alexandria, Egypt, from February 2024 to September 2024. The minimum sample size was set at 200 individuals. From Fisher’s formula for sample size calculation using the prevalence from the previous study,

$$ {\text{n = }}{{{\mathrm{Z}}^{{\mathrm{2}}} {\mathrm{p}}\left( {100 - {\mathrm{p}}} \right)} \mathord{\left/ {\vphantom {{{\mathrm{Z}}^{{\mathrm{2}}} {\mathrm{p}}\left( {100 - {\mathrm{p}}} \right)} {{\mathrm{d}}^{{\mathrm{2}}} }}} \right. \kern-\nulldelimiterspace} {{\mathrm{d}}^{{\mathrm{2}}} }} $$

where n is the sample size, z is the statistic corresponding to the decided level of confidence, in this case, 95% with a z value of 1.96, p is the prevalence obtained from the previous study, and d is the amount of error that can be tolerated by the study, in this case, 5%.

## Inclusion Criteria

Both sexes were aged more than 18 years old and radiologically diagnosed with OA, activity-related knee pain, in addition to signs and symptoms indicative of knee OA, including knee pain, by examination revealed knee swelling, stiffness, and sufficient synovial effusion to allow arthrocentesis for fluid analysis [39].

## Data and Sample Collection

A structured questionnaire was used to collect sociodemographic data, causes of OA, duration of illness, stage of arthritis (acute/chronic), clinical symptoms, radiological findings, and type of treatment received. Knee effusion is classified into 4 scales: **0** represents a normal physiologic amount of fluid, **1** indicates a mild effusion, where fluid remains confined within the retropatellar space, **2** corresponds to a moderate effusion, characterized by a slight convexity of the suprapatellar bursa, and **3** signifies a severe effusion, evident by capsular distention [40]. Although SF aspiration has a theranostic role - a therapeutic measure that provides significant knee pain relief and aids in the diagnosis [41]—it was performed only after obtaining formal consent from each participant. Arthrocentesis was performed, and SF samples were collected from the affected knees in sterile, labeled Falcon tubes and transported to the Tropical Health Lab at the High Institute of Public Health, Alexandria University, for processing.

## Synovial Fluid Analysis for Parasitosis

Synovial fluid samples were aspirated, and freshly prepared thin films were spread and stained with various stains, including modified Ziehl-Neelsen (MZN), trichrome, and Quick-Hot-Gram-Chromotrope (QHGC) stains, to detect the parasites [42–44]. The remaining samples were centrifuged, and the sediment of each was inoculated in Jones’ media for detecting *Blastocystis* sp. [45].

## Synovial Fluid Analysis for Biomarkers (TNF-α and MMP9)

Synovial fluid samples were stored at -20℃ and then thawed for molecular analysis. Both TNF-α and MMP-9 levels were quantified using Elabscience^®^ ELISA Kits (Catalog nos. E-EL-H0109 and E-EL-H6075, respectively).

### Statistical Analysis

The data were input into a computer and analyzed via the IBM SPSS software package version 20.0. **(**Armonk, NY: IBM Corp**)**. Categorical data are presented as numbers and percentages. The relationship between two qualitative variables was examined by the **chi-square test**; alternatively, **Fisher’s exact correction** test was applied when more than 20% of the cells had expected counts less than 5. A risk assessment was carried out using the relative risk and odds ratio to quantify the risk associated with the independent variable. The Kolmogorov-Smirnov test was used to assess the normality of continuous data. The significance of the acquired results was assessed at the 5% level.

## Results

Table 1 shows that the highest percentages of osteoarthritic patients enrolled in the present study were females, those aged ≥ 60 years, and those not working (60%, 59%, and 58%, respectively). Concerning synovial fluid appearance, turbid samples constituted a high percentage (82%), while the mechanical causes of arthritis constituted 85% of the studied samples. Regarding clinical symptoms, osteoarthritic patients presented chronic disease and tenderness (86.5%, and 85.5%, respectively). In terms of injectable therapy, OP treated with corticosteroids (CST) or non-corticosteroids (non-CST), such as hyaluronic acid (HA) and platelet-rich plasma (PRP), presented similar rates (50%).

**Table 1:** Distribution of Osteoarthritic patients according to some host factors, clinical features:

Host factors		*N* (200)	%
Gender	Males	80	40.0
	Females	120	60.0
Age (years)	< 60	82	41.0
	≥ 60	118	59.0
Job	Not work	116	58.0
	Employee	64	32.0
	Player	20	10.0
*Clinical features*			
Duration of illness (years)	< 5	100	50.0
	≥ 5	100	50.0
Stage of OA*	Acute	27	13.5
	Chronic	173	86.5
Causes of OA**	Mechanical	170	85.0
	Traumatic	17	8.5
	Secondary causes	13	6.5
Tenderness	No	29	14.5
	Yes	171	85.5
Severity of Effusion	Mild	54	27.0
	Moderate	100	50.0
	Severe	46	23.0
Appearance of SF	Clear	36	18.0
	Turbid	164	82.0
Flexion attitude	No	28	14.0
	Mild	91	45.5
	Moderate	57	28.5
	Severe	24	12.0
Injectable therapies	No	10	5.0
	HA	60	30.0
	PRP	30	15.0
	CST	73	36.5
	HA & CST	10	5.0
	PRP & CST	10	5.0
	HA, PRP, & CST	7	3.5

*Acute pain is pain that lasts for a specific time, has a specific cause, and has a protective function. Chronic pain is long-lasting, and its cause may be elusive, making it harder to treat

**Fig. 1:**
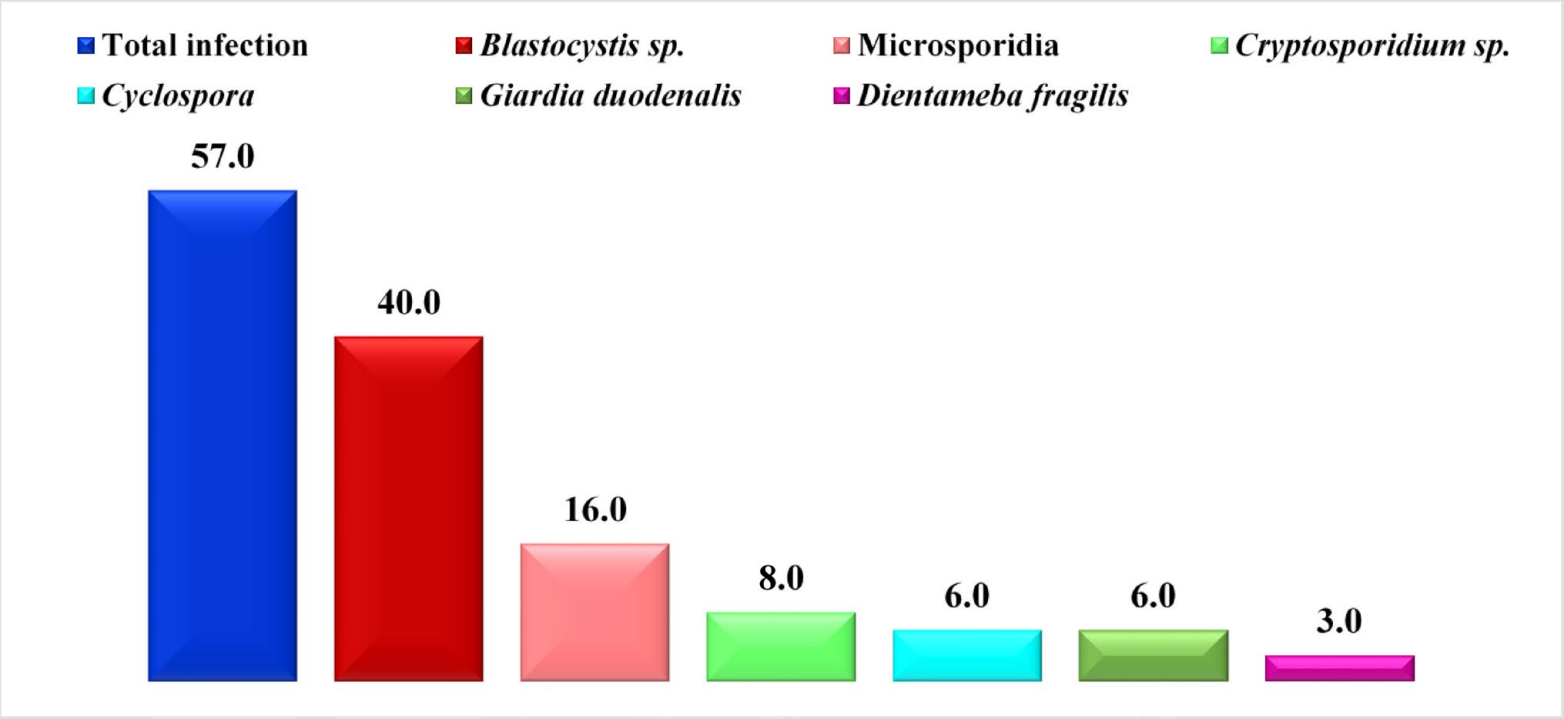
Percentages of synovial parasitosis among OA patients

**Fig. 2:**
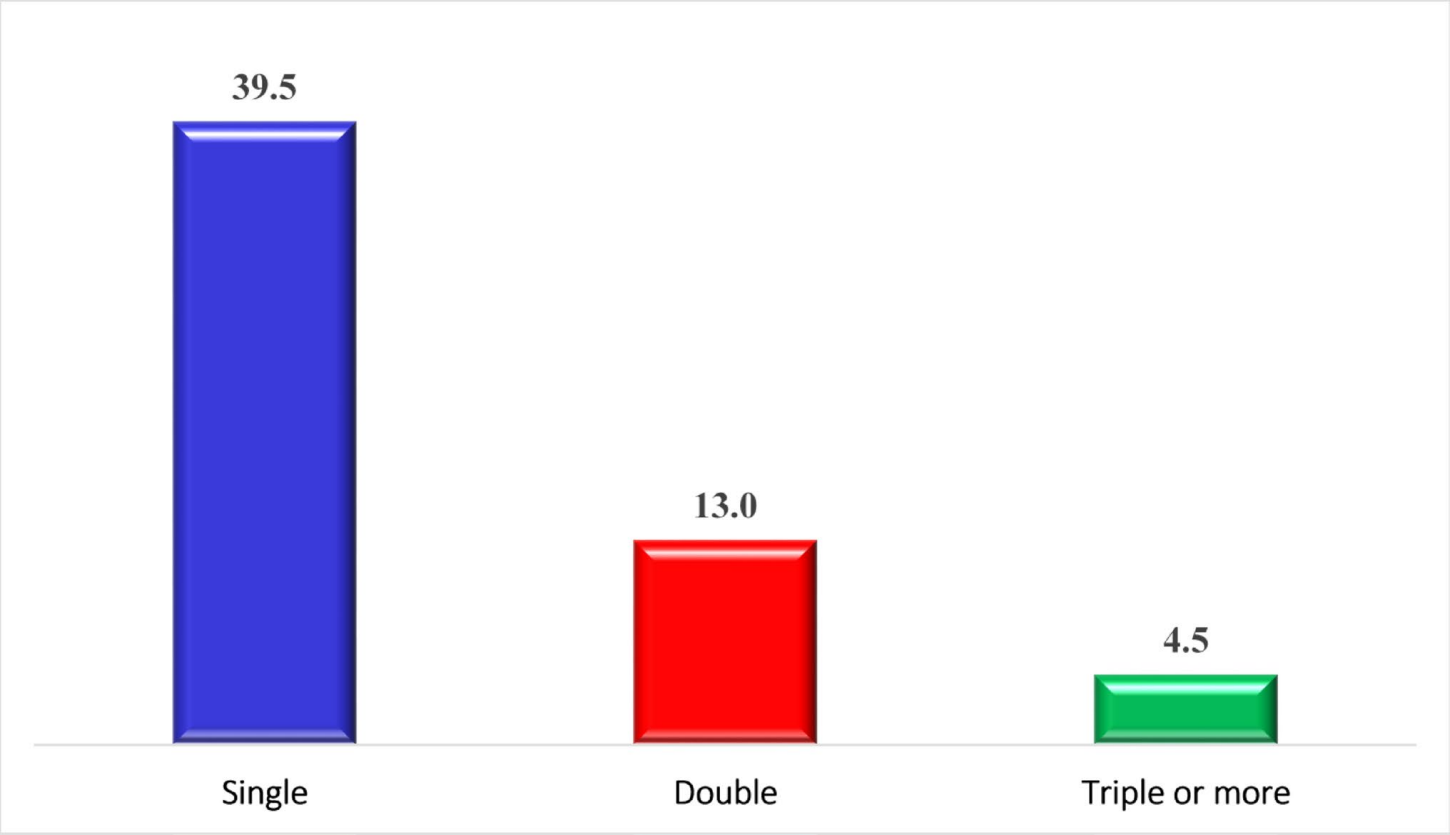
Percentages of synovial parasitosis multiplicity among infected patients

Of 200 OA patients, 114 (57%) had synovial parasitosis (SP). *Blastocystis* sp. had the highest rate of infection in synovial fluid (40%), followed by microsporidia and *Cryptosporidium* sp. (16% and 8%, respectively). Similarly, lower rates (6%) were detected for both *Cyclospora cayetanensis* (*C. cayetanensis*) and *Giardia duodenalis* (*G duodenalis*), whereas *Dientameba fragilis* (*D. fragilis*) presented the lowest rate of infection (3%) (Fig. 1). Infected osteoarthritic patients had the highest rate of single infection, followed by double and triple infections (39.5%, 13%, and 4.5%, respectively), (Fig. 2).

To achieve the study, aim of examining synovial parasitosis and its associated biomarker profiles in osteoarthritic patients, we first assessed the distribution of parasitosis concerning corticosteroid injectable therapies and host factors (Table 2). We then investigated whether biomarker levels, specifically MMP-9 and TNF-α, differed between infected and non-infected patients across the same host factors (Tables 3 and 4). This approach allowed us to explore both the clinical correlates of parasitosis and its impact on inflammatory biomarker expression, thereby providing a comprehensive view of the parasitosis–biomarker relationship in osteoarthritis.

**Table 2:** Association of corticosteroid injectable therapies with synovial parasitosis and host factors among Osteoarthritic patients:

Parameters	Infected patients *N* = 114					
	None CST *N* = 33 (%)	CST *N* = 81 (%)		O. *R*.	C.I 95%	*p*
*Gender*						
Male	14 (42.4)	35 (43.2)		1.033	0.427–2.195	1.00
Female	19 (57.6)	46 (58.8)				
*Age*						
< 60	16 (48.5)	30 (37.0)		1.6	0.71–3.63	0.296
≥ 60	17 (51.5)	51 (63.0)				
*Appearance of SF*						
Clear	11 (33.3)	7 (8.6)		5.29	1.83–15.26	0.003*
Turbid	22 (66.7)	74 (91.4)				
*Duration of illness*						
< 5 years	22 (66.7)	30 (37.0)		3.4	1.45–7.98	0.007*
≥ 5 years	11 (33.3)	51 (63.0)				
*Stage of OA*						
Acute	6 (18.2)	10 (12.3)		1.58	0.523–4.76	0.552
Chronic	27 (81.8)	71 (87.7)				
Knee pain						
Unilateral	21 (63.6)	47 (58.0)		1.266	0.549–2.92	0.676
Bilateral	12 (36.4)	34 (42.0)				
*Severity of effusion*						
Mild	5(15.2)	22 (27.1)		–	–	0.207
Moderate	23 (69.7)	42 (51.9)				
Severe	5(15.2)	17 (21.0)				
Biomarkers	Mean ± SD.	Mean ± SD.				
TNF (pg/ml)	40.9 ± 10.96	37.26 ± 11.78				0.12
MMP9 (ng/ml)	0.99 ± 0.44	0.93 ± 0.35				0.408

OR: Odd`s ratio C.I.: Confidence interval LL: Lower limit UL: Upper limit

χ^2^: Chi square test

* p*:* p*-value for comparing the two studied groups

*: Statistically significant at *p* ≤ 0.05

Table 2 examines the relationship between corticosteroid injectable therapies and the occurrence of synovial parasitosis across different host factors in osteoarthritic patients. Overall, there were no significant differences in parasitic positivity between CST-treated and non-CST–treated males or females [43.2% vs. 42.4% and 58.8% vs. 57.6%, respectively]. Among patients younger than 60 years, those treated with CST had slightly lower rates of parasitosis than those on non-CST therapy (37% vs. 48.5%), whereas in patients aged 60 years or older, CST treatment was associated with higher parasitosis rates (63% vs. 51.5%); however, these differences were not statistically significant. Turbid synovial fluid samples from CST-treated patients showed a significantly higher frequency of parasitosis than those from non-CST–treated patients (91.4% vs. 66.7%, *P* = 0.003). A longer duration of illness was also associated with significantly higher rates of parasitosis in the CST group compared to the non-CST group (63% vs. 33.3%, *P* = 0.007). Across other host factors, including OA chronicity (87.7% vs. 81.8%), laterality of pain (42% vs. 36.4%), and effusion grade (15.2% vs. 27.1%, 69.6 vs.51.9, and 15.2% vs. 21.0%, respectively), CST-treated patients tended to show higher parasitosis frequencies, but the differences were not statistically significant. Biomarker analysis indicated that CST-treated patients had slightly lower mean levels of TNF-α and MMP-9 compared to non-CST–treated patients (37.26 ± 11.78 vs. 40.9 ± 10.96 and 0.93 ± 0.35 vs. 0.99 ± 0.44, respectively), but again without statistical significance. Taken together, these findings suggest that corticosteroid therapy does not exert a protective effect against synovial parasitosis and that infection risk may persist across different host factors.

Table 3 demonstrates that osteoarthritic patients with synovial parasitosis consistently exhibited significantly higher MMP-9 levels compared to non-infected patients across all examined host factors. Both infected males and females had markedly elevated MMP-9 concentrations compared with their non-infected counterparts [(0.90 ± 0.34 vs. 0.48 ± 0.11 and 0.98 ± 0.40 vs. 0.47 ± 0.10, respectively, *P* < 0.001)]. Similarly, patients younger than 60 years and those aged 60 years or older who were infected showed significantly higher MMP-9 levels than non-infected patients in the same age groups [(0.84 ± 0.35 vs. 0.38 ± 0.07) and (1.01 ± 0.038 vs. 0.54 ± 0.07), respectively, *P* < 0.001]. Infected patients with either clear or turbid synovial fluid [(0.97 ± 0.39 vs. 0.51 ± 0.07) and (0.94 ± 0.38 vs. 0.47 ± 0.11), respectively], shorter or longer illness duration [(0.95 ± 0.42 vs. 0.48 ± 0.11) and (0.95 ± 0.33 vs. 0.48 ± 0.09), respectively], acute or chronic OA [(0.91 ± 0.47 vs. 0.45 ± 0.10) AND (0.95 ± 0.36 vs. 0.48 ± 0.11), respectively], unilateral or bilateral pain [0.97 ± 0.39 vs. 0.49 ± 0.11 and 0.90 ± 0.35 vs. 0.46 ± 0.10, respectively], and different degrees of effusion also exhibited significantly higher MMP-9 concentrations than non-infected patients [0.76 ± 0.20 vs. 0.48 ± 0.09, 1.02 ± 0.43 vs. 0.46 ± 0.10, and 0.95 ± 0.30 vs. 0.49 ± 0.13, respectively, *P* < 0.001]. These trends were consistent among both CST-treated and non-CST–treated patients [(0.99 ± 0.44 vs. 0.48 ± 0.11) and (0.93 ± 0.35 vs. 0.47 ± 0.07), respectively]. Collectively, these findings highlight MMP-9 elevation as a robust biomarker signature associated with synovial parasitosis in osteoarthritic patients, independent of demographic or clinical host factors.

**Table 3:** Association between MMP-9 profiles in osteoarthritic patients suffering from parasitosis stratified by host factors:

	MMP9 (ng/ml)						U	*p*
	Non parasitic infection			Total parasitic infection				
	No	Mean ± SD		No	Mean ± SD			
*Gender*								
Male	31	0.48 ± 0.11		49	0.90 ± 0.34		80.00^*^	< 0.001^*^
Female	55	0.47 ± 0.10		65	0.98 ± 0.40		176.00^*^	< 0.001^*^
Age years								
< 60	36	0.38 ± 0.07		46	0.84 ± 0.35		12.50^*^	< 0.001^*^
≥ 60	50	0.54 ± 0.07		68	1.01 ± 0.38		143.00^*^	< 0.001^*^
*Appearance of SF*								
Clear	18	0.51 ± 0.07		18	0.97 ± 0.39		8.500^*^	< 0.001^*^
Turbid	68	0.47 ± 0.11		96	0.94 ± 0.38		324.00^*^	< 0.001^*^
*Duration of illness*								
< 5	48	0.48 ± 0.11		52	0.95 ± 0.42		119.00^*^	< 0.001^*^
≥ 5	38	0.48 ± 0.09		62	0.95 ± 0.33		134.00^*^	< 0.001^*^
*Stage of arthritis*								
Acute	11	0.45 ± 0.10		16	0.91 ± 0.47		6.000^*^	< 0.001^*^
Chronic	75	0.48 ± 0.11		98	0.95 ± 0.36		380.0^*^	< 0.001^*^
Knee Pain								
Unilateral	52	0.49 ± 0.11		68	0.97 ± 0.39		169.50^*^	< 0.001^*^
Bilateral	34	0.46 ± 0.10		46	0.90 ± 0.35		79.00^*^	< 0.001^*^
*Severity of Effusion*								
Mild	27	0.48 ± 0.09		27	0.76 ± 0.20		28.50^*^	< 0.001^*^
Moderate	35	0.46 ± 0.10		65	1.02 ± 0.43		77.00^*^	< 0.001^*^
Severe	24	0.49 ± 0.13		22	0.95 ± 0.30		44.50^*^	< 0.001^*^
*Injectable therapies*								
Non-CST	67	0.48 ± 0.11		33	0.99 ± 0.44		124.00^*^	< 0.001^*^
CST	19	0.47 ± 0.07		81	0.93 ± 0.35		42.00^*^	< 0.001^*^

SD: Standard deviation U: Mann Whitney test

*p*: *p* value for comparing between Non parasitic infection and Total parasitic infection

^*^: Statistically significant at *p* ≤ 0.05

Table 4 shows that osteoarthritic patients with synovial parasitosis had consistently higher TNF-α levels compared to non-infected patients, regardless of host factors. Both infected males and females displayed significantly elevated TNF-α compared with their non-infected counterparts [(37.95 ± 10.86 vs. 20.08 ± 3.14 and 38.63 ± 12.25 vs. 19.09 ± 2.79, respectively, *P* < 0.001)]. Infected patients younger than 60 years and those aged 60 years or older exhibited significantly higher TNF-α levels than non-infected patients in the same age categories [(35.31 ± 11.50 vs. 17.45 ± 1.96) and (40.39 ± 11.34 vs. 20.88 ± 2.69), respectively, *P* < 0.001]. Elevated TNF-α was also evident among infected patients with clear or turbid synovial fluid [(40.27 ± 9.49 vs. 19.21 ± 2.32) and (37.98 ± 11.99 vs. 19.51 ± 3.10), respectively, *P* < 0.001], illness duration of less than or greater than five years [(38.45 ± 11.89 vs. 19.52 ± 2.91) and (38.25 ± 11.50 vs. 19.35 ± 3.02), respectively, *P* < 0.001], acute or chronic OA [(39.28 ± 10.28 vs. 19.56 ± 2.15) and (38.19 ± 11.87 vs.19.43 ± 3.05), respectively, *P* < 0.001], unilateral or bilateral pain [38.58 ± 11.89 vs. 19.63 ± 2.89 and 37.98 ± 11.35 vs. 19.17 ± 3.04, respectively, *P* < 0.001], and varying degrees of effusion [35.03 ± 8.86 vs. 19.45 ± 2.95, 40.67 ± 12.05 vs. 19.03 ± 2.63, and 35.51 ± 12.19 vs. 20.05 ± 3.36, respectively, *P* < 0.001]. Similar patterns were observed in both CST-treated and non-CST–treated groups [(40.99 ± 10.96 vs. 19.52 ± 3.04) and (37.26 ± 11.78 vs. 19.19 ± 2.63), respectively, *P* < 0.001]. These consistent elevations suggest that TNF-α is an important proinflammatory biomarker associated with synovial parasitosis in osteoarthritic patients, independent of demographic or clinical factors

**Table 4:** Association between TNF-α profiles in osteoarthritic patients suffering from parasitosis stratified by host factors:

	TNF-α (pg/ml)						U	*p*
	Non-parasitic infection			Total parasitic infection				
	No	Mean ± SD		No	Mean ± SD			
*Gender*								
Male	31	20.08 ± 3.14		49	37.95 ± 10.86		124.00^*^	< 0.001^*^
Female	55	19.09 ± 2.79		65	38.63 ± 12.25		313.00^*^	< 0.001^*^
*Age years*								
< 60	36	17.45 ± 1.96		46	35.31 ± 11.50		173.00^*^	< 0.001^*^
≥ 60	50	20.88 ± 2.69		68	40.39 ± 11.34		229.00^*^	< 0.001^*^
*Appearance of SF*								
Clear	18	19.21 ± 2.32		18	40.27 ± 9.49		18.00^*^	< 0.001^*^
Turbid	68	19.51 ± 3.10		96	37.98 ± 11.99		611.50^*^	< 0.001^*^
*Duration of illness*								
< 5	48	19.52 ± 2.91		52	38.45 ± 11.89		202.00^*^	< 0.001^*^
≥ 5	38	19.35 ± 3.02		62	38.25 ± 11.50		213.00^*^	< 0.001^*^
*Stage of arthritis*								
Acute	11	19.56 ± 2.15		16	39.28 ± 10.28		0.000^*^	< 0.001^*^
Chronic	75	19.43 ± 3.05		98	38.19 ± 11.87		726.00^*^	< 0.001^*^
Knee Pain								
Unilateral	52	19.63 ± 2.89		68	38.58 ± 11.89		295.00^*^	< 0.001^*^
Bilateral	34	19.17 ± 3.04		46	37.98 ± 11.35		138.00^*^	< 0.001^*^
*Severity of Effusion*								
Mild	27	19.45 ± 2.95		27	35.03 ± 8.86		48.00^*^	< 0.001^*^
Moderate	35	19.03 ± 2.63		65	40.67 ± 12.05		176.50^*^	< 0.001^*^
Severe	24	20.05 ± 3.36		22	35.51 ± 12.19		79.50^*^	< 0.001^*^
*Injectable therapies*								
Non-CST	67	19.52 ± 3.04		33	40.99 ± 10.96		100.50^*^	< 0.001^*^
CST	19	19.19 ± 2.63		81	37.26 ± 11.78		151.00^*^	< 0.001^*^

SD: Standard deviation U: Mann Whitney test

*p*: *p* value for comparing between Non parasitic infection and Total parasitic infection

^*^: Statistically significant at *p* ≤ 0.05

Table 5 shows that the infected OPs had higher TNF-α and MMP9 levels than the noninfected patients did [38.34 ± 1.01 vs. 19.45 ± 0.32 and 0.95 ± 0.35 vs. 0.48 ± 0.01, respectively], with highly statistically significant differences (*P* < 0.001). Concerning the multiplicity of SPs and biomarkers among OPs. The present study revealed highly statistically significant differences (*P* < 0.001) between TNF-α and MMP9 levels as the number of parasites infected increased, starting from single infections (33.94 ± 1.19 and 0.82 ± 0.02), double infections (48.42 ± 1.56 and 1.18 ± 0.11), and triple or more parasites (47.80 ± 0.47 and 1.4 ± 0.01).

**Table 5:** Multiplicity of synovial parasitosis and biomarkers among Osteoarthritic patients:

SP	Biomarkers *N* = 200				
	TNF-α	*P*-value	MMP-9	*P*-value	
*Total parasitic infection*					
-Ve	19.45 ± 0.32	0.00*	0.48 ± 0.01		0.00*
+ve	38.34 ± 1.01		0.95 ± 0.35		
*Multiplicity of SP*					
None	19.45 ± 0.32	0.00*	0.48 ± 0.01		0.00*
Single	33.94 ± 1.19		0.82 ± 0.02		
Double	48.42 ± 1.56		1.18 ± 0.11		
Triple or more	47.80 ± 0.47**		1.4 ± 0.01**		

Data are presented as mean ± S.E.

*p*:* p*-value for comparing the two studied groups

*: Statistically significant at *p* ≤ 0.05

## Discussion

Our findings demonstrate that synovial parasitosis in osteoarthritic patients is not only influenced by host and therapeutic factors, such as corticosteroid use, but is also consistently associated with elevated inflammatory biomarkers (MMP-9 and TNF-α). This highlights parasitosis as a clinically relevant condition with distinct biomarker profiles, directly supporting our study aim of characterizing both the occurrence of synovial parasitosis and its related biomarker signatures in osteoarthritis.

Infections caused by bacteria, viruses, fungi, or other uncommon pathogens spread from the body to large joints, such as the knee, through the bloodstream, resulting in septic arthritis. Physiological changes in SF are due to inflammation, trauma, and microbial invasion; thus, SF aspiration and analysis are essential for theragnosis, especially when patients present with acutely painful joints, potential infection, inflammation, or noninflammatory effusion [16, 46]. In our study, OA caused by mechanical causes constituted the highest percentage (85%), followed by traumatic causes (8.5%), and secondary causes (6.5%). Thus, we studied synovial parasitosis among osteoarthritic patients because of the scarcity of previous studies.

Our findings revealed that the total SP among OPs was 57%, and the most prevalent parasites were *Blastocystis* sp., microsporidia, and *Cryptosporidium* sp. (40%, 16%, and 8%, respectively), followed by *C. cayetanensis* and *G. duodenalis* (6%), and *D. fragilis* (3%). The single infection rate was the highest, followed by the double and triple infections (39.5%, 13%, and 4.5%, respectively).

To our knowledge, only a few studies have published case reports on septic arthritis and parasitic infection, in addition to other studies that enrolled patients with rheumatoid arthritis and parasitic infection; however, no studies have considered Ops, especially in synovial fluid. In 1990, Lee et al., [37] detected *Blastocystis* sp. in SF via trichrome stain from the left knee of a seronegative rheumatoid arthritis patient aged 29 years. Lee and colleagues attributed this to her immunosuppression, which may have resulted in systemic dissemination of *Blastocystis* sp. from the gastrointestinal tract with resultant infective arthritis. In 2012, Tejera et al. [38] reported reactive arthritis due to *Blastocystis* sp. among a 45-year-old Spanish woman with monoarthritis of the left knee and heel pain. In 2019, Hussein et al. [47] reported that the prevalence of parasitic rheumatism was 48% among out-clinic patients suffering from unexplained rheumatic pain at Suez Canal University Hospital. The most commonly detected intestinal parasites were *C. cayetanensis*, *G. lamblia*, *Blastocystis* sp., and *E. histolytica* (32%, 24%, 20%, and 8%, respectively). Microsporidia, *Schistosoma mansoni*,* Ascaris lumbricoides*, and *Strongyloides stercoralis* (*S. stercoralis*) were detected at lower rates (4%) each. Among mixed infections, 32% were triple infections, and 50% of patients were infected with *Cryptosporidium*,* C. cayetanensis*,* E. histolytica/dispar*, or *S. stercoralis.* In 2022, Guzmán-Guzmán et al. [48] reported that 50% of patients suffering from rheumatoid arthritis had intestinal protozoa, 22.2% of whom were infected with *Blastocystis* sp., and 27.8% had coinfection with *Blastocystis* sp. with other protozoa.

Recent studies suggest that *G. duodenalis* and *Blastocystis* sp. may exhibit extraintestinal dissemination under certain conditions, including immune suppression. *Blastocystis*, previously considered a gut commensal, has been implicated in systemic inflammation and detected in extraintestinal sites [37, 38, 47, 48]. In 2020, Escutia-Guzman and colleagues [49] reported that while extra enteric infections caused by *Blastocystis* sp. are rare, they identified the vacuolar stages of this microorganism via Papanicolaou staining in cervical samples of a 47-year-old Mexican woman, who presented with slight vaginal itching during a routine gynecological examination. This study highlights the remarkable ability of *Blastocystis* sp. to colonize new niches, even in patients with minimal symptoms [49]. Similarly, Prodeus and colleagues [50] detected typical amoeboid and vacuolar stages of *Blastocystis* sp. in a 64-year-old Russian female patient who developed a liver abscess. Additionally, it was detected in the splenic cyst fluid of a 22-year-old Brazilian patient [51]. Similarly, *G. duodenalis* has been reported in extraintestinal environments, possibly due to immune complex-mediated dissemination or direct translocation [52]. These observations are consistent with our findings of these parasites in synovial fluid, underscoring the need for further molecular and experimental research.

Although locally injectable corticosteroid therapy reduces pain secondary to inflammation in symptomatic osteoarthritis [53], it is contraindicated in cases of superficial or deep infection [54]. Our findings revealed a link between SP and injectable treatments in patients with osteoarthritis. Compared with those who did not receive CST, osteoarthritic patients who received CST had greater SP. This was observed in males and females (43.2% vs. 42.4% and 58.8% vs. 57.6%, respectively), those aged ≥ 60 years (63% vs. 51.5%), those with chronic OA (87.7% vs. 81.8%), and those with bilateral knee pain (42% vs. 36.4%). Moreover, infected osteoarthritic patients treated with CST showed lower means of TNF-α and MMP9 biomarkers than did those not treated with CST [(37.26 ± 11.78 vs. 40.9 ± 10.96) and (0.93 ± 0.35 vs. 0.99 ± 0.44), respectively], with insignificant statistical differences (*P >* 0.05). In contrast, compared with non-CST-treated patients, osteoarthritic patients treated with CST had high SP among those with turbid SF (91.4% vs. 66.7%), a duration of illness ≥ 5 years (63% vs. 33.3%), and swelling of the knees (91.4% vs. 51.5%) were significantly difference (*P* < 0.01). Previous studies revealed that several predisposing risk factors, such as age, malnutrition, micronutrient deficiency, living or traveling in endemic areas, comorbidities, immunomodulatory therapy, including corticosteroids, azathioprine, and methotrexate, and biologic therapy such as anti-TNF agents increase the risk for parasitic infection, resulting in the suppression of innate immunity and dissemination of the parasitic infection [55–58].

Our findings revealed an association between SP and biomarkers, including TNF-α and MMP9, among osteoarthritic patients. Compared with males, infected females exhibited higher TNF-α and MMP-9 levels. Those with turbid synovial fluid, bilateral knee pain, or those treated with corticosteroids showed lower—but statistically insignificant—levels of these biomarkers (*P* > 0.05). In contrast, infected osteoarthritic patients aged ≥ 60 years and those with effusion presented significantly higher levels of both TNF-α and MMP9 and [(40.39 ± 1.38 and 1.014 ± 0.046) and (39.59 ± 1.20 and 0.98 ± 0.04), respectively] (*P* < 0.05). In 2023, Kumar revealed that the levels of biomarkers such as MMP9 and TNF-α in the SF were positively correlated with the severity of OA in the knee, age, and duration of illness. However, no significant correlation was found between the levels of MMP9 and TNF-α and gender, knee inflammation, morning stiffness, or duration of illness [59]. Uncontrolled MMP activity during the late or chronic stages of infection results in host tissue damage and even death [25].

Although corticosteroid therapy (CST) may increase susceptibility to parasitic infection through suppression of immune surveillance, our findings showed elevated TNF-α and MMP-9 levels among patients with synovial parasitosis. This likely reflects an ongoing inflammatory response to parasitic invasion rather than a direct proinflammatory effect of CST. Similar mechanisms have been described in other infections, where immune suppression predisposes to infection, but inflammation is driven by the pathogen once infection is established (Mehta et al., 2020) [60].

In 2022, Guzmán-Guzmán et al. [48] revealed that parasites play an important role in immune response modulation; hence, protozoal infection promotes an increase in TNF-α in patients with rheumatoid arthritis. Our results revealed that compared with noninfected patients, the infected OPs had high levels of TNF-α and MMP9 [(38.34 ± 1.01 vs. 19.45 ± 0.32) and (0.95 ± 0.35 vs. 0.48 ± 0.011), respectively], with highly statistically significant differences (*P* < 0.001). Concerning the multiplicity of SPs and biomarkers among osteoarthritic patients. The present study revealed highly statistically significant differences (*P* < 0.001) in TNF-α and MMP9 levels with increasing the multiplicity of infection, including single infection (33.94 ± 1.19 and 0.82 ± 0.024), double infection (48.42 ± 1.56 and 1.18 ± 0.11), and three or more parasites (47.80 ± 0.47 and 1.4 ± 0.01). Guzmán-Guzmán et al., [48] reported that the total incidence of protozoal infection was 50% among patients suffering from RA; 27% had a single infection, and 22.2% had coinfection. *Blastocystis* sp. and *Endolimax nana* had the highest rates of infection. Guzmán-Guzmán and colleagues reported that coinfection was related to increased levels of TNF-α, mainly in patients receiving antirheumatic treatment. Protozoal infection is associated with increased intestinal permeability in patients with RA; therefore, infection, coinfection, and an abundance of intestinal protozoa should be clinically screened, as they may be associated with clinical disease variability [48]. This study has certain limitations that should be acknowledged. The relatively small sample size may limit the generalizability of the findings and increase the potential for sampling bias. Furthermore, the lack of molecular confirmation represents a methodological constraint that could affect the accuracy of species identification and data interpretation. Recognizing these limitations provides a balanced perspective on the study’s outcomes and highlights areas for improvement in future research.

## Conclusions

Our study highlights a significant prevalence of SP among OA patients (57%) and the highest rate of *Blastocystis* sp. Notably, patients treated with CST showed a higher synovial parasitosis (SP) compared with those not treated with CST. This study suggests that elevated TNF-α and MMP9 levels could be biomarkers for identifying patients at risk of more severe disease progression due to SP. Further research should be conducted using molecular techniques, along with studies utilizing antiparasitic treatment among infected OA patients.

## Supplementary Information

Below is the link to the electronic supplementary material.

Supplementary Material 1

Supplementary Material 2

## Data Availability

The data generated or analyzed during this study are included in this article.

## Abbreviations

CST: Corticosteroid treatment
C. cayetanensis: *Cyclospora cayetanensis*
D. fragilis: *Dientameba fragilis*
ELISA: Enzyme-linked immunosorbent assay
ECM: Extracellular matrix
G duodenalis: *Giardia duodenalis *
IFN-γ: Interferon‐gamma
IL: Interleukin
MMP: Matrix metalloproteases
NSAIDs: Nonsteroidal anti-inflammatory drugs
OP: Osteoarthritic patients
OA: Osteoarthritis
RA: Rheumatic arthritis
S. stercoralis: *Strongyloides stercoralis*
SF: Synovial fluid
SP: Synovial parasitosis
TNF: Tumor necrosis factor
