# Supplementary material for: Synovial Parasitosis and Inflammatory Biomarker Profiles in Osteoarthritis: Associations with Host and Therapeutic Factors
Source: Acta Parasitol. 2025 Dec 18;71(1):11. doi: 10.1007/s11686-025-01180-2 (PMC12715035; doi:10.1007/s11686-025-01180-2)
Supplement: Supplementary file 1 — Supplementary Material 1 [file 11686_2025_1180_MOESM1_ESM.docx]

**Figures**

**Figure 1: Percentages of synovial parasitosis among OA patients**

**Figure 2: Percentages of synovial parasitosis multiplicity among infected patients**
