# Supplementary material for: Synovial Parasitosis and Inflammatory Biomarker Profiles in Osteoarthritis: Associations with Host and Therapeutic Factors
Source: Acta Parasitol. 2025 Dec 18;71(1):11. doi: 10.1007/s11686-025-01180-2 (PMC12715035; doi:10.1007/s11686-025-01180-2)
Supplement: Supplementary file 2 — Supplementary Material 2 [file 11686_2025_1180_MOESM2_ESM.docx]

**Table 1: Distribution of osteoarthritic patients according to some host factors, clinical features:**

| **Host factors** | | **N (200)** | **%** |
| --- | --- | --- | --- |
| **Gender** | Males | 80 | 40.0 |
|  | Females | 120 | 60.0 |
| **Age (years)** | <60 | 82 | 41.0 |
|  | ≥ 60 | 118 | 59.0 |
| **Job** | Not work | 116 | 58.0 |
|  | Employee | 64 | 32.0 |
|  | Player | 20 | 10.0 |
| **Clinical Features** | | | |
| **Duration of illness (years)** | <5 | 100 | 50.0 |
|  | ≥5 | 100 | 50.0 |
| **Stage of OA*** | Acute | 27 | 13.5 |
|  | Chronic | 173 | 86.5 |
| **Causes of OA**** | **Mechanical** | 170 | 85.0 |
|  | **Traumatic** | 17 | 8.5 |
|  | **Secondary causes** | 13 | 6.5 |
| **Tenderness** | No | 29 | 14.5 |
|  | Yes | 171 | 85.5 |
| **Severity of Effusion** | Mild | 54 | 27.0 |
|  | Moderate | 100 | 50.0 |
|  | Severe | 46 | 23.0 |
| **Appearance of SF** | Clear | 36 | 18.0 |
|  | Turbid | 164 | 82.0 |
| **Flexion attitude** | No | 28 | 14.0 |
|  | Mild | 91 | 45.5 |
|  | Moderate | 57 | 28.5 |
|  | Severe | 24 | 12.0 |
| **Injectable therapies** | No | 10 | 5.0 |
|  | HA | 60 | 30.0 |
|  | PRP | 30 | 15.0 |
|  | CST | 73 | 36.5 |
|  | HA & CST | 10 | 5.0 |
|  | PRP & CST | 10 | 5.0 |
|  | HA, PRP, & CST | 7 | 3.5 |

*****Acute pain is pain that lasts for a specific time, has a specific cause, and has a protective function. Chronic pain is long-lasting, and its cause may be elusive, making it harder to treat.

****Mechanical causes:** Heavy activities, heavy loads, prolonged loads, vertical loads, and prolonged sitting

**Traumatic causes:** Subjected to knee stress (valgus injury), direct trauma, and falling down

**Secondary causes:** Rheumatological conditions (e.g., rheumatoid arthritis contributing to osteoarthritis), septic arthritis, and post-operation (e.g., joint surgeries leading to accelerated osteoarthritis)

**HA:** Hyaluronic acid **PRP:** Platelet-rich plasma **CST:** Corticosteroid Treatment

**Table 2: Association of Corticosteroid injectable therapies with synovial parasitosis and host factors among osteoarthritic patients:**

| **Parameters** | **Infected patients N=114** | |  | | | |
| --- | --- | --- | --- | --- | --- | --- |
|  | **None CST**  **N=33 (%)** | **CST**  **N= 81 (%)** | | **O. R.** | **C.I 95%** | **p** |
| **Gender** | | | | | | |
| **Male** | 14 (42.4) | 35 (43.2) | | 1.033 | 0.427 – 2.195 | 1.00 |
| **Female** | 19 (57.6) | 46 (58.8) | |  |  |  |
| **Age** | | | | | | |
| **<60** | 16 (48.5) | 30 (37.0) | | 1.6 | 0.71 – 3.63 | 0.296 |
| **≥60** | 17 (51.5) | 51 (63.0) | |  |  |  |
| **Appearance of SF** | | | | | | |
| **Clear** | 11 (33.3) | 7 (8.6) | | 5.29 | 1.83 – 15.26 | 0.003* |
| **Turbid** | 22 (66.7) | 74 (91.4) | |  |  |  |
| **Duration of illness** | | | | | | |
| **< 5 years** | 22 (66.7) | 30 (37.0) | | 3.4 | 1.45 – 7.98 | 0.007* |
| **≥ 5 years** | 11 (33.3) | 51 (63.0) | |  |  |  |
| Stage of OA | | | | | | |
| **Acute** | 6 (18.2) | 10 (12.3) | | 1.58 | 0.523 – 4.76 | 0.552 |
| **Chronic** | 27 (81.8) | 71 (87.7) | |  |  |  |
| Knee pain | | | | | | |
| **Unilateral** | 21 (63.6) | 47 (58.0) | | 1.266 | 0.549 – 2.92 | 0.676 |
| **Bilateral** | 12 (36.4) | 34 (42.0) | |  |  |  |
| Severity of Effusion | | | | | | |
| **Mild** | 5(15.2) | 22 (27.1) | | ---- | ---- | 0.207 |
| **Moderate** | 23 (69.6) | 42 (51.9) | |  |  |  |
| **Severe** | 5(15.2) | 17 (21.0) | |  |  |  |
| Biomarkers | **Mean ± SD.** | **Mean ± SD.** | |  |  |  |
| **TNF (pg/ml)** | 40.9 ±10.96 | 37.26 ±11.78 | |  |  | 0.12 |
| **MMP9 (ng/ml)** | 0.99 ± 0.44 | 0.93 ± 0.35 | |  |  | 0.408 |

OR: Odd`s ratio C.I.: Confidence interval LL: Lower limit UL: Upper Limit

χ^2^: **Chi square test**

p: p-value for comparing the two studied groups

*: Statistically significant at p ≤ 0.05

**Table (3): Association between MMP-9 profiles in osteoarthritic patients suffering from parasitosis stratified by host factors:**

|  | **MMP9 (ng/ml)** | | | | | | **U** | **p** |
| --- | --- | --- | --- | --- | --- | --- | --- | --- |
|  | **Non parasitic infection** | | | **Total parasitic infection** | | |  |  |
|  | **No.** | **Mean ± SD.** | **Median  (Min. – Max.)** | **No.** | **Mean ± SD.** | **Median  (Min. – Max.)** |  |  |
| **Gender** |  |  |  |  |  |  |  |  |
| Male | 31 | 0.48 ± 0.11 | 0.50 (0.30 – 0.64) | 49 | 0.90 ± 0.34 | 0.86 (0.49 – 2.27) | 80.00^*^ | <0.001^*^ |
| Female | 55 | 0.47 ± 0.10 | 0.48 (0.30 – 0.67) | 65 | 0.98 ± 0.40 | 0.85 (0.47 – 2.04) | 176.00^*^ | <0.001^*^ |
| **Age years** |  |  |  |  |  |  |  |  |
| < 60 | 36 | 0.38 ± 0.07 | 0.39 (0.30 – 0.57) | 46 | 0.84 ± 0.35 | 0.76 (0.47 – 2.27) | 12.50^*^ | <0.001^*^ |
| ≥ 60 | 50 | 0.54 ± 0.07 | 0.55 (0.40 – 0.67) | 68 | 1.01 ± 0.38 | 0.93 (0.49 – 2.04) | 143.00^*^ | <0.001^*^ |
| **Appearance of SF** |  |  |  |  |  |  |  |  |
| Clear | 18 | 0.51 ± 0.07 | 0.53 (0.31 – 0.64) | 18 | 0.97 ± 0.39 | 0.84 (0.57 – 1.82) | 8.500^*^ | <0.001^*^ |
| Turbid | 68 | 0.47 ± 0.11 | 0.48 (0.30 – 0.67) | 96 | 0.94 ± 0.38 | 0.86 (0.47 – 2.27) | 324.00^*^ | <0.001^*^ |
| **Duration of illness** |  |  |  |  |  |  |  |  |
| <5 | 48 | 0.48 ± 0.11 | 0.48 (0.30 – 0.66) | 52 | 0.95 ± 0.42 | 0.78 (0.54 – 2.27) | 119.00^*^ | <0.001^*^ |
| ≥5 | 38 | 0.48 ± 0.09 | 0.49 (0.30 – 0.67) | 62 | 0.95 ± 0.33 | 0.89 (0.47 – 1.96) | 134.00^*^ | <0.001^*^ |
| **Stage of arthritis** |  |  |  |  |  |  |  |  |
| Acute | 11 | 0.45 ± 0.10 | 0.43 (0.30 – 0.64) | 16 | 0.91 ± 0.47 | 0.71 (0.54 – 2.27) | 6.000^*^ | <0.001^*^ |
| Chronic | 75 | 0.48 ± 0.11 | 0.49 (0.30 – 0.67) | 98 | 0.95 ± 0.36 | 0.88 (0.47 – 2.04) | 380.0^*^ | <0.001^*^ |
| **Knee Pain** |  |  |  |  |  |  |  |  |
| Unilateral | 52 | 0.49 ± 0.11 | 0.50 (0.30 – 0.66) | 68 | 0.97 ± 0.39 | 0.88 (0.49 – 2.27) | 169.50^*^ | <0.001^*^ |
| Bilateral | 34 | 0.46 ± 0.10 | 0.48 (0.30 – 0.67) | 46 | 0.90 ± 0.35 | 0.85 (0.47 – 1.96) | 79.00^*^ | <0.001^*^ |
| **Severity of Effusion** |  |  |  |  |  |  |  |  |
| Mild | 27 | 0.48 ± 0.09 | 0.50 (0.31 – 0.67) | 27 | 0.76 ± 0.20 | 0.72 (0.54 – 1.43) | 28.50^*^ | <0.001^*^ |
| Moderate | 35 | 0.46 ± 0.10 | 0.48 (0.30 – 0.66) | 65 | 1.02 ± 0.43 | 0.90 (0.49 – 2.27) | 77.00^*^ | <0.001^*^ |
| Severe | 24 | 0.49 ± 0.13 | 0.56 (0.30 – 0.66) | 22 | 0.95 ± 0.30 | 0.92 (0.47 – 1.42) | 44.50^*^ | <0.001^*^ |
| **Injectable therapies** |  |  |  |  |  |  |  |  |
| Non-CST | 67 | 0.48 ± 0.11 | 0.49 (0.30 – 0.67) | 33 | 0.99 ± 0.44 | 0.89 (0.47 – 2.27) | 124.00^*^ | <0.001^*^ |
| CST | 19 | 0.47 ± 0.07 | 0.48 (0.34 – 0.58) | 81 | 0.93 ± 0.35 | 0.85 (0.49 – 2.04) | 42.00^*^ | <0.001^*^ |

SD: **Standard deviation** **U: Mann Whitney test**

p: p value for comparing between **Non parasitic infection** and **Total parasitic infection**

*: Statistically significant at p ≤ 0.05

**Table (4): Association between TNF-α profiles in osteoarthritic patients suffering from parasitosis stratified by host factors:**

|  | **TNF-α (pg/ml)** | | | | | | **U** | **p** |
| --- | --- | --- | --- | --- | --- | --- | --- | --- |
|  | **Non-parasitic infection** | | | **Total parasitic infection** | | |  |  |
|  | **No.** | **Mean ± SD.** | **Median (Min. – Max.)** | **No.** | **Mean ± SD.** | **Median (Min. – Max.)** |  |  |
| **Gender** |  |  |  |  |  |  |  |  |
| Male | 31 | 20.08 ± 3.14 | 20.06 (15.63 – 26.17) | 49 | 37.95 ± 10.86 | 38.32 (13.70 – 62.75) | 124.00^*^ | <0.001^*^ |
| Female | 55 | 19.09 ± 2.79 | 18.78 (15.54 – 27.17) | 65 | 38.63 ± 12.25 | 40.02 (12.77 – 62.10) | 313.00^*^ | <0.001^*^ |
| **Age years** |  |  |  |  |  |  |  |  |
| < 60 | 36 | 17.45 ± 1.96 | 16.69 (15.60 – 23.70) | 46 | 35.31 ± 11.50 | 37.57 (12.77 – 62.75) | 173.00^*^ | <0.001^*^ |
| ≥ 60 | 50 | 20.88 ± 2.69 | 21.09 (15.54 – 27.17) | 68 | 40.39 ± 11.34 | 42.73 (13.70 – 62.10) | 229.00^*^ | <0.001^*^ |
| **Appearance of SF** |  |  |  |  |  |  |  |  |
| Clear | 18 | 19.21 ± 2.32 | 19.07 (15.99 – 23.57) | 18 | 40.27 ± 9.49 | 40.07 (15.71 – 55.82) | 18.00^*^ | <0.001^*^ |
| Turbid | 68 | 19.51 ± 3.10 | 19.10 (15.54 – 27.17) | 96 | 37.98 ± 11.99 | 38.74 (12.77 – 62.75) | 611.50^*^ | <0.001^*^ |
| **Duration of illness** |  |  |  |  |  |  |  |  |
| <5 | 48 | 19.52 ± 2.91 | 18.88 (15.63 – 26.17) | 52 | 38.45 ± 11.89 | 39.00 (14.39 – 62.75) | 202.00^*^ | <0.001^*^ |
| ≥5 | 38 | 19.35 ± 3.02 | 19.35 (15.54 – 27.17) | 62 | 38.25 ± 11.50 | 39.84 (12.77 – 56.96) | 213.00^*^ | <0.001^*^ |
| **Stage of arthritis** |  |  |  |  |  |  |  |  |
| Acute | 11 | 19.56 ± 2.15 | 19.45 (15.63 – 22.20) | 16 | 39.28 ± 10.28 | 39.10 (24.81 – 62.75) | 0.000^*^ | <0.001^*^ |
| Chronic | 75 | 19.43 ± 3.05 | 18.89 (15.54 – 27.17) | 98 | 38.19 ± 11.87 | 39.46 (12.77 – 62.10) | 726.00^*^ | <0.001^*^ |
| **Knee Pain** |  |  |  |  |  |  |  |  |
| Unilateral | 52 | 19.63 ± 2.89 | 19.31 (15.60 – 26.17) | 68 | 38.58 ± 11.89 | 39.89 (13.70 – 62.75) | 295.00^*^ | <0.001^*^ |
| Bilateral | 34 | 19.17 ± 3.04 | 18.80 (15.54 – 27.17) | 46 | 37.98 ± 11.35 | 38.66 (12.77 – 56.96) | 138.00^*^ | <0.001^*^ |
| **Severity of Effusion** |  |  |  |  |  |  |  |  |
| Mild | 27 | 19.45 ± 2.95 | 19.40 (15.54 – 27.17) | 27 | 35.03 ± 8.86 | 35.55 (15.71 – 49.81) | 48.00^*^ | <0.001^*^ |
| Moderate | 35 | 19.03 ± 2.63 | 18.73 (15.63 – 25.89) | 65 | 40.67 ± 12.05 | 42.68 (12.77 – 62.75) | 176.50^*^ | <0.001^*^ |
| Severe | 24 | 20.05 ± 3.36 | 20.23 (15.60 – 26.17) | 22 | 35.51 ± 12.19 | 35.01 (13.70 – 50.95) | 79.50^*^ | <0.001^*^ |
| **Injectable therapies** |  |  |  |  |  |  |  |  |
| Non-CST | 67 | 19.52 ± 3.04 | 18.98 (15.54 – 27.17) | 33 | 40.99 ± 10.96 | 41.75 (16.07 – 62.75) | 100.50^*^ | <0.001^*^ |
| CST | 19 | 19.19 ± 2.63 | 19.22 (15.87 – 23.70) | 81 | 37.26 ± 11.78 | 38.45 (12.77 – 61.45) | 151.00^*^ | <0.001^*^ |

SD: **Standard deviation** **U: Mann Whitney test**

p: p value for comparing between **Non parasitic infection** and **Total parasitic infection**

*: Statistically significant at p ≤ 0.05

**Table 5: Multiplicity of synovial parasitosis and biomarkers among osteoarthritic patients:**

| **SP** | **Biomarkers N=200** | | | | |
| --- | --- | --- | --- | --- | --- |
|  | **TNF-α** | **P-value** | **MMP-9** | **P-value** | |
| **Total parasitic infection** | | | | | |
| **-Ve** | 19.45 ± 0.32 | 0.00* | 0.48 ± 0.01 | | 0.00* |
| **+ve** | 38.34 ± 1.01 |  | 0.95 ± 0.35 | |  |
| **Multiplicity of SP** | | | | | |
| **None** | 19.45 ± 0.32 | 0.00* | 0.48 ± 0.01 | | 0.00* |
| **Single** | 33.94 ± 1.19 |  | 0.82 ± 0.02 | |  |
| **Double** | 48.42 ± 1.56 |  | 1.18 ± 0.11 | |  |
| **Triple or more** | 47.80 ± 0.47** |  | 1.4 ± 0.01** | |  |

**Data are presented as mean ± S.E.**

p: p-value for comparing the two studied groups

*: Statistically significant at p ≤ 0.05
